# Differential Antecedents and Consequences of Affective and Cognitive Ruminations

**DOI:** 10.3390/ijerph191811452

**Published:** 2022-09-12

**Authors:** Huaying Lin, Xinwen Bai

**Affiliations:** 1 Institute of Psychology, Chinese Academy of Sciences, Beijing 100101, China; 2 Department of Psychology, University of Chinese Academy of Sciences, Beijing 100049, China

**Keywords:** rumination, affective and cognitive ruminations, challenge and hindrance stressors, positive and negative affects, learning behavior, cognitive job insecurity, interpersonal conflict, information processing perspective

## Abstract

Adopting the information processing perspective, the current study aims to investigate the differential effects of affective and cognitive ruminations on individuals’ affective states and learning behavior, and to further explore their differential mediating roles in transmitting effects of challenge and hindrance stressors on affect and behavior. A two-wave survey, in which stressors and ruminations were measured in the first wave and affective states and learning behavior were measured in the second, was conducted to obtain responses from 410 employees. As expected, affective and cognitive ruminations were differently associated with challenge stressors (i.e., cognitive job insecurity) and hindrance stressors (i.e., interpersonal conflict), and yielded different effects in terms of positive affect, negative affect, and learning behavior. Specifically, the results showed that: (a) cognitive job insecurity was significantly and positively related to cognitive rumination, while interpersonal conflict was significantly and positively related to affective rumination; (b) affective and cognitive ruminations were significantly associated with positive or negative affect, but in the exact opposite direction; (c) cognitive rumination, but not affective rumination, was significantly related to learning behavior; and (d) cognitive rumination mediated the effect of cognitive job insecurity on positive affect and learning behavior, while affective rumination mediated the effect of interpersonal conflict on negative affect. The current study contributes to the literature on rumination by introducing a new perspective, and sheds new light on the understanding of how and why affective and cognitive ruminations may lead to different affective states and behaviors.

## 1. Introduction

When experiencing drastic change, especially in the face of negative stressful events, people often have difficulty suppressing their repetitive thoughts, thus leading to ruminative thinking [1,2]. Nolen-Hoeksema [3] defines rumination as an individual’s repetitive thinking about negative emotions and their antecedents and consequences. This view has been widely adopted by many researchers [4], and a large number of studies have consistently shown that ruminative thinking can lead to a range of negative consequences [5,6], including emotional disorders such as depression [7], suicidal ideation [8], eating disorders [9], impulsive and aggressive behaviors [10], and low cognitive flexibility [11] or problem-solving efficiency [12].

In fact, rumination does not always result in negative effects. The consequences of rumination primarily depend on whether the nature of the event an individual is concerned about is positive or negative, whether the individual is focusing on the positive or negative aspect of the same stressful event, or whether the rumination is executed in a positive or negative manner [13,14]. If ruminative thinking focuses on positive rather than negative affect [15,16,17], or a person focuses on positive rather than only negative personal experiences [18], or rumination can be carried out in an active way [19], rather than just in a passive or intrusive manner [20,21], rumination may also bring about positive effects.

Although researchers generally acknowledge that rumination can bring about positive consequences [13], there are few relevant empirical studies. More importantly, existing studies on rumination have two major limitations. Firstly, most studies have been devoted to the scrutiny of depressive rumination that always results in negative affect [5], and less attention has been paid to other forms of rumination. It should be noted that depressive rumination is essentially a type of affective rumination that is oriented towards negative affect [16], and usually demonstrates negative effects [13]. By contrast, people are also engaging in cognitive rumination, i.e., consciously trying to avoid emotional disturbance so that they can focus on the problem itself to find solutions [22,23]. Preliminary findings have suggested that cognitive rumination can have a positive effect, although this positive effect is unstable [24]. Therefore, it is worth exploring whether the effects of affective rumination and cognitive rumination can be differentiated.

As a second limitation, the few studies that have demonstrated a positive effect of rumination have predominantly focused on individuals who have experienced major traumatic stress. It has been shown that rumination can sometimes help people achieve personal growth after undergoing major trauma [20,25]. However, most stressful events that people experience in their daily life and work are not severe enough to cause traumatic stress. The question of how daily stressful events stimulate rumination and ultimately lead to positive or negative outcomes through the transmission of different ruminations remains an underexplored topic in the literature [24]. Although it has attracted some preliminary interest from a few researchers [23,26], the question of whether there are differential consequences between affective and cognitive ruminations still requires more empirical exploration. The current study aims to fill the gap by examining the differential antecedents and consequences of affective and cognitive ruminations. Specifically, we aim to reveal how two types of stressful events people often encounter in their daily life and work will yield differential effects on an individual’s affect and learning behavior through the differential mediating effects of the two forms of ruminations.

### 1.1. Differential Effects of Affective and Cognitive Ruminations

The defining feature of rumination is continuous repetitive thinking, a common response occurring after people undergo negative stress events [1,2]. Rumination can be divided into different types according to the contents and patterns of the repetitive thinking involved. Nolen-Hoeksema [3] defined rumination as an individual’s repetitive thinking about the manifestations, causes, effects, and significance of his own negative affect. Immersive rumination and reflective rumination, as two key dimensions, are both forms of repetitive thinking about emotional feelings. The former only focuses on passive ruminations thinking, while the latter focuses on active and purposeful cognitive adjustment [27]. This is typical of uncontrolled repetitive thinking about negative affect and is also known as depressive rumination because it is common in patients with depression. However, people can also have ruminative thinking with regard to positive affect [15,16]. Depressive rumination often leads to negative consequences [5], while rumination with regard to positive affect has positive effects [17]. In addition to emotional states, personal experiences and stressful events themselves are objects of rumination as well [18]. An individual’s rumination on negative experiences or events, which usually leads to adverse outcomes, is considered to be negative rumination. Rumination on positive experiences and events, which may bring about positive effects, is called positive rumination [18,26]. According to the degree of self-control over ruminative thinking, some scholars have divided rumination into two categories: intrusive rumination and active rumination [21]. Intrusive rumination intrudes into an individual’s cognitive processes under uncontrolled, unexpected conditions after major stressful events, such as natural disasters, major illness, or other personal traumatic experiences. Active rumination is intentional, targeted, repetitive thinking proactively initiated by an individual. Intrusive rumination immerses individuals in stressful events and is difficult to escape, so that they cannot achieve post-traumatic growth. Active rumination can help individuals effectively adjust their cognition, adapt to unfavorable environments, and finally achieve self-growth [20,28].

Upon experiencing stressful events, individual’s repetitive information processing also involves two modes: affective processing and cognitive processing. Employing the information processing view, Cropley and colleagues propose a classification framework in the workplace that distinguishes ruminative thinking between affective and cognitive ruminations [22,23]. Affective rumination focuses on one’s own emotional experience, and is a typical form of affective processing. On the contrary, cognitive rumination is problem-solving oriented. Driven by the mode of cognitive processing, those performing cognitive rumination are trying to come up with solutions [23]. Following this classification framework [22,23], the current paper also divides rumination into affective rumination and cognitive rumination. Affective rumination refers to intrusive, diffuse, repetitive thinking that triggers negative affect; cognitive rumination refers to continuous unemotional thinking for the purpose of obtaining solutions.

This view is consistent with the dual-process theory of information processing [29]. The dual-process theory holds that the difference between affective rumination and cognitive rumination lies in the modes of information processing involved. The former focuses on affective processing, while the latter focuses on cognitive processing. This further results in differences between the two forms of rumination in multiple stages of information processing. First, the information and cues involved in the processing are different. Affective rumination focuses on understanding the emotions and states that occur after undergoing stress, while cognitive rumination focuses on trying to clarify how stressful events challenge one’s existing belief system [14]. Second, the reference standards for information processing are different. Affective rumination is mainly based on subjective experience and attitudes for decision-making and judgment, while cognitive rumination is more based on the objective characteristics of stressful events [30]. Third, the dynamic processes of the two types of information processing are different. Affective rumination is a typical form of hot processing, characterized by a rapid judgment and decision-making process based on perceptual experience. By comparison, cognitive rumination is a form of slow, cold processing mainly based on rational judgment, which tries to eliminate emotional disturbance [31]. Finally, there are significant differences between the two types of information processing in terms of goals and time orientation. Affective rumination is aimed at the past and the present, with the major goal of feeling one’s current emotional experience and trying to understand the cause of the emotion. Cognitive rumination is also aimed at the past, such as understanding the cause of the problem. More importantly, however, it is oriented toward the future, with the core goal of thinking about a plan to solve the problem [13].

This study expects that it is precisely because of the above-mentioned differences in information processing between affective rumination and cognitive rumination that the two will present different effects at the emotional and behavioral levels when an individual undergoes stressful events. Specifically, this study intends to explore whether the two types of rumination have different effects on the three outcome variables of negative affect, positive affect, and learning behavior. Negative and positive affect are important indicators measuring subjective well-being [32], which can sensitively capture changes in an individual’s psychological state when they are faced with external stress [33]. Therefore, they are suitable indicators that can reveal the effects of affective and cognitive ruminations. In addition, whether an individual can learn from stress, setbacks, and failures is key to whether they can truly overcome adversity and achieve growth [34]. This study adopts the suggestion of Watkins [13]. In addition to focusing on the results at the emotional level, this study will also expand to the behavioral level to explore how the two types of rumination affect an individual’s learning behavior.

Nolen-Hoeksema [3] proposed that depressive rumination is a typical form of affective rumination. A number of studies employing this framework have consistently indicated that affective rumination has negative effects on an individual’s affect and coping behavior [5]. This is because affective rumination, which repeatedly focuses on negative affect, will not only prolong the duration of the negative affect, but also continuously increase its intensity, eventually leading to emotional disorders such as depression and anxiety [1]. It is not difficult to see that such affective rumination will exacerbate negative affect and reduce an individual’s positive affect. In terms of learning behavior, the amount of learning behavior depends on whether an individual has learning opportunities, motivation, and ability [35]. From the perspective of information processing, the reason why affective rumination significantly reduces problem-solving efficiency is that continuous immersion in negative affect will narrow an individual’s cognitive range, reduce sensitivity to external environmental stimuli, and cause cognitive flexibility and concentration to decline, so that the individual is more likely to show an avoidance orientation rather than an approach orientation [5]. Thus, it is inferred that affective rumination is not conducive for an individual to spot learning opportunities, reducing learning motivation and cognitive ability, thus reducing learning behaviors in turn. By contrast, through cognitive rumination, an individual shifts attention to how to formulate solutions and action plans. This not only helps to get rid of negative affect [23] and convert attention to positive affect [15,16,17], but also helps to improve thinking flexibility [36], and more likely helps the individual find their own gaps, thus enabling them to be more motivated and more able to devote themselves to learning. Therefore, the following hypotheses are proposed:

**Hypothesis** **1.** *Affective rumination leads to negative consequences. Specifically, affective rumination is positively associated with negative affect (H1a), and negatively associated with both positive affect (H1b) or learning behavior (H1c).*

**Hypothesis** **2.** *Cognitive rumination produces positive consequences. Specifically, cognitive rumination is negatively associated with negative affect (H2a) and positively associated with positive affect (H2b) or learning behavior (H2c).*

### 1.2. Differential Mediating Effects of Affective and Cognitive Ruminations

Ruminative thinking is a response stimulated by external pressure factors [5]. Most studies have only explored how a single factor, such as major natural disasters [20] or an individual’s major traumatic experiences [37], stimulates different types of ruminative thinking. However, in daily work and life, an individual often faces multiple stresses of different natures at the same time. For example, Cavanaugh et al. [38] proposed a very influential classification framework that divides stressors into hindrance stressors and challenge stressors. Hindrance stressors in the workplace can manifest as many forms, such as organizational politics, bureaucratic culture, ambiguous responsibilities and role conflicts, and interpersonal conflict, among others. Hindrance stressors are detrimental to an individual’s development. Challenge stressors can also bring a sense of stress to an individual, but an individual may be able to achieve growth and make progress by overcoming the difficulties. Typical challenge stressors include heavy workload, a tight work schedule, large work responsibilities, a wide range of work, and high requirements for one’s ability in the process of undertaking tasks. Several studies have shown that hindrance stressors produce only negative effects, such as a sense of stress, reduction of work motivation and performance, work burnout, etc. Challenge stressors can also bring about stress and burnout, but can also stimulate work motivation and improve work efficiency and work engagement [39,40].

Recently, researchers have also begun to explore whether different types of stressors affect ruminative thinking in different ways with reference to the hindrance-challenge stressor framework. Frone [18] surveyed agent representatives at a call center as subjects, and the results indicated that positive work experiences (e.g., fair distribution, opportunities to make friends at work, and pleasant work contents) stimulate positive rumination, while negative work experiences (e.g., extremely high job requirements, job contents leading to emotional distress) stimulate negative rumination. In the Chinese context, some scholars [26] have adopted the positive-negative rumination scale developed by Frone [18] to directly explore how hindrance stressors and challenge stressors stimulate the two types of rumination. The results indicate that there is a significant contrast between the effect modes of the two types of stressors on the two types of rumination. Challenge stressors trigger active rumination and reduce insomnia through the mediating effect of active rumination. Conversely, hindrance stressors trigger negative rumination and lead to insomnia through the mediating effect of negative rumination. In addition, hindrance stressors negatively predict positive rumination. Van Laethem et al. [41] found that challenge stressors could stimulate affective rumination and reduce job performance through the mediating effect of affective rumination. However, this hypothesis has not been supported, as the path by which the authors expected hindrance stressors to stimulate affective rumination is not significant.

This is actually consistent with the dual-process theory of information processing [29]. Research has demonstrated that events of a different nature will activate different modes of information processing. Specifically, while events that result in salient emotional reactions usually activate an affective mode, those that can stimulate people to reflect elicit a cognitive mode [30]. Consequently, in the former situation, people are usually dealing with their personal feelings about the events, while they tend to focus on the objective characteristics of stressful events in the latter situation [14]. Therefore, we propose that hindrance stressors will activate an affective mode of information processing, since they only produce negative emotional reactions, and that challenge stressors will activate a cognitive mode because they can stimulate in-depth reflection [39,40].

It should be noted that all of these studies only measured general stressors rather than targeting a certain specific stressful event. Given that rumination is generally triggered by a specific negative stimulus, such as a major traumatic experience [37] or being treated unfairly by others [42], this study holds that if a typical challenge constituting a hindrance stressor is accurately measured, that will better reveal its differential effects on affective rumination and cognitive rumination. Interpersonal conflict is the most representative type of hindrance stressor [38] and is also the most common kind of stressor, as each individual is a member of a social group, and interpersonal conflict will inevitably occur in interpersonal interactions. When an interpersonal conflict occurs, once handled improperly, it will throw people into negative affect such as a sense of stress and burnout [43] and even make people feel helpless [44]. It is reasonable to expect that an individual’s negative affect will be highlighted by interpersonal conflict. In such a case, the individual will be likely to engage in affective rumination.

When initially proposing their stressor classification framework, Cavanaugh et al. [38] considered job insecurity as a type of typical hindrance stressor. However, through intensive research, researchers have found that job insecurity actually involves both cognitive and affective ingredients, and correspondingly there exists a distinction between cognitive job insecurity and affective job insecurity [45]. The former is an individual’s perception that his job position, responsibilities, etc. are being threatened; the latter is an individual’s emotional response after he perceives his job being threatened, including alertness, worry, anxiety, fear, etc. as the representative reactions. From the classification framework of challenge-hindrance stressors, affective job insecurity is a typical hindrance stressor. However, when an individual perceives the uncertainty of his future job, a sense of crisis about job prospects will be triggered. Although this sense of crisis can lead to a sense of stress, it also prompts people to make future plans, objectively providing them with opportunities for self-improvement and development. Therefore, cognitive job insecurity is a challenge stressor. It is precisely under the driving force of cognitive job insecurity that one is more likely to carefully analyze the opportunities and challenges in the workplace, reflect on one’s strengths and weaknesses, and formulate action plans, and thus one is more likely to perform cognitive rumination.

Therefore, by integrating the dual-process theory of information processing [29] and the hindrance-challenge stressors framework [38], we speculate that interpersonal conflict and cognitive job insecurity will yield differential effects on affective and cognitive ruminations. Thus, we propose Hypotheses 3 and 4.

**Hypothesis** **3.** *Interpersonal conflict is positively associated with affective rumination.*

**Hypothesis** **4.** *Cognitive job insecurity is positively associated with cognitive rumination.*

Based on the above hypotheses, we also expect that the two types of stressors will have different effects on the two types of affect and learning behavior through the differential mediating effects of the two forms of rumination. Thus, Hypotheses 5 and 6 are proposed.

**Hypothesis** **5.** *Affective rumination mediates the effect of interpersonal conflict on negative affect (H5a), positive affect (H5b), or learning behavior (H5c).*

**Hypothesis** **6.** *Cognitive rumination mediates the effect of cognitive job insecurity on negative affect (H6a), positive affect (H6b), or learning behavior (H6c).*

**Figure 1:**
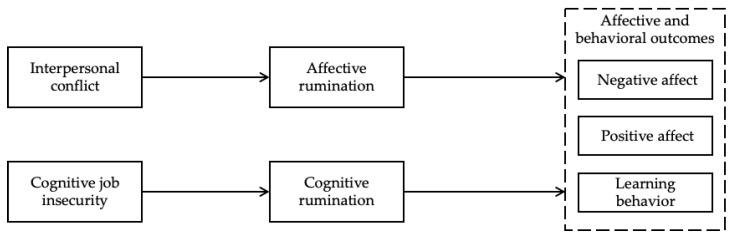
Theoretical Model.

To summarize, the current study aims (a) to investigate the differential effects of affective and cognitive ruminations on individuals’ affective states and learning behavior, and (b) to explore their differential mediating roles in transmitting effects of challenge and hindrance stressors on these affective and behavioral outcomes. Figure 1 depicts our theoretical model.

## 2. Materials and Methods

### 2.1. Procedures

The current study aims to reveal the differential antecedents and consequences of affective and cognitive ruminations by collecting survey data. We distributed the survey questionnaire via Credamo (www.credamo.com), a reliable Chinese online survey platform that is similar to Qualtrics and widely used to recruit participants in China. To reduce common method variance, we relied on the two-wave survey strategy to collect data [46]. The first survey was conducted at the end of November, 2021. In the first survey, the independent variables (i.e., the two types of stressors) and the mediator variables (i.e., affective and cognitive ruminations), as well as demographic information, were measured. A total of 517 full-time employees were invited to voluntarily participate in the survey. Among them, seventeen participants failed the attention-check test and were dropped from further analysis; the remaining 500 with valid responses were retained, resulting in a response rate of 96.7%. Two weeks later (i.e., in mid-November 2021), a second survey was conducted to measure dependent variables (i.e., positive affect, negative affect, and learning behavior). Only the 500 respondents who had provided valid responses in the first survey were invited, and a total of 420 respondents participated. Ten of them failed the attention-check test, and 410 returned with valid questionnaires, resulting in a response rate of 95.6% for the second survey. As a result, a total of 410 participants (with a response rate of 82.0%) gave valid responses in both surveys.

### 2.2. Sample

Among the final sample that consisted of 410 participants, 164 were males (40%) and 246 were females (60%). Of these, 173 (42.2%) were under 30 years old, 212 (51.7%) were 31–40 years old, and 25 (6.1%) were 41–60 years old. Fifty (12.19%) possessed a technical college degree or below, 288 (70.2%) had an undergraduate degree, and 72 (17.6%) had a postgraduate degree. A majority of 351 respondents (85.6%) were working in the private sector, and the rest of the 59 respondents (14.4%) were from the public service sector. Their mean job tenure was 8.54 years (with a standard deviation of 5.52).

### 2.3. Measures

All scales have been successfully used in early studies under the Chinese context. Stated otherwise, all items were responded to on the five-point Likert scale.

Cognitive and affective ruminations. We used Cropley et al.’s [47] ten-item scale to measure affective rumination and cognitive rumination, which has demonstrated good reliability in the Chinese context [42]. As the original items were limited to working scenarios, we slightly adapted the wording to cover events at work and in life. Sample items are “I will be annoyed at thinking about previous issues after the event” (affective rumination) and “I will reflect on how to improve my ability and performance” (cognitive rumination). The *α* coefficients for the two dimensions in the current study were 0.87 and 0.76, respectively.

Interpersonal conflict. The eight-item interpersonal conflict scale developed by Giebels and Janssen [48] is widely applied and often used by Chinese researchers [49]. Sample items are “I have a personal conflict with others” and “Because of previous conflict with others, I feel that I do not have harmonious relationships with them”. The coefficient α was 0.79 in the current study.

Cognitive job insecurity. The job insecurity scale developed by Huang et al. [45] in the Chinese context was used. There were four items for the dimension of cognitive job insecurity, a sample item being “How sure are you that your knowledge and skills will still be applicable in the next five years?” The *α* coefficient was 0.82 in the current study.

Positive and negative effects. The scale developed by Warr [50], which had good reliability in the Chinese context [51], was used to measure positive and negative affect, with each involving eight items. Representative items for positive affect are “enthusiastic”, “delighted”, “excited”, and “inspired”; representative items for negative affect are “nervous”, “low-spirited”, “anxious”, and “depressed”. Affective states were measured in the second survey. To better capture the lagged effect of ruminations on respondents’ affective states, respondents were asked to answer by reflecting on their actual feelings during the past two weeks (i.e., since the first survey). The *α* coefficients for the two dimensions in the current study were 0.87 and 0.86, respectively.

Learning behavior. The learning behavior scale developed by Bezuijen et al. [52], which had demonstrated good reliability in the Chinese context [53], was used in the current study. It consists of eight items that mainly reflected how frequently an individual engages in learning of all sorts. Sample items are “I try to expand my knowledge and skills” and “I will discuss and ask others how to make progress.” Similarly, learning behavior was measured in the second survey, and the respondents were asked to answer questions according to their actual situation in the past two weeks. The *α* coefficient was 0.80.

### 2.4. Analytical Strategies

Confirmatory factor analysis (CFA) was first conducted to confirm the validity of variable measurement and to examine to what extent the common method variance exists [46]. Following Baron and Kenny’s seminal paper [54], a series of hierarchical regressions were conducted to formally test all of the hypotheses. Relying on Hayes’ PROCESS macro [55], we further constructed bias-corrected confidence intervals to test hypotheses involving mediating effects.

## 3. Results

### 3.1. Measurement Model and Common Method Variance

A series of CFAs were performed to test whether the measurement model for key variables met the requirements. An item parceling strategy was adopted considering a larger number of items for several variables (e.g., interpersonal conflict, learning behavior, positive affect, and negative affect, each consisting of eight items), with each including four parcels [56]. CFA results were shown in Table 1.

As expected, the hypothesized seven-factor model in which each of the seven variables was independent had a satisfactory fit with the data (χ^2^ (384) = 870.51, *p* < 0.001, CFI = 0.92, TLI = 0.90, RMSEA = 0.056, SRMR = 0.06). To examine the discriminant validity of the measurements, we further constructed a series of alternative six-factor models in which two of these variables were combined into one factor. As can be seen from Table 1, each of the six-factor models fitted poorly with the data, and was worse than that of the hypothesized seven-factor model (i.e., all Δχ^2^ were significant at the level of *p* < 0.001), indicating an adequate discriminant validity for our measurement model. Moreover, Harman’s single-factor model in which all items were on a single factor fitted poorly, indicating that the common method variance would not significantly affect the results in our two-wave survey data [46].

### 3.2. Descriptive Statistics

Table 2 shows the descriptive statistics for the key variables. It can be seen that affective rumination was significantly correlated with interpersonal conflict and negative affect in the positive direction, and significantly correlated to cognitive job insecurity, positive affect and learning behavior in the negative direction. Cognitive rumination was significantly correlated with negative affect in the negative direction, and significantly correlated with cognitive job insecurity, positive affect and learning behavior in the positive direction. Furthermore, interpersonal conflict was significantly correlated with negative affect in the positive direction and significantly correlated with positive affect and learning behavior in the negative direction; cognitive job insecurity was significantly correlated with these variables in the opposite direction. This is consistent with our hypotheses, providing preliminary support for hypothesis testing. Next, we will rely on regression analysis to formally test all of the hypotheses.

### 3.3. Hypothesis Testing

Since the tests of Hypotheses 5 and 6 (i.e., the mediating effects of the two forms of rumination) were embedded with the tests of Hypotheses 1 and 2 (i.e., the effects of two types of rumination on the three outcome variables) as well as Hypotheses 3 and 4 (i.e., the effects of the two types of stressors on the two types of rumination), all hypotheses were tested using hierarchical regression analyses proposed by Baron and Kenny [55]. In all regression models, gender, age, and job tenure were included as control variables. The results are shown in Table 3 and Table 4, respectively. As shown in Models 3, 6, and 9 in Table 4, the effects of affective rumination and cognitive rumination on three outcome variables were exactly in opposite directions, which were consistent with Hypotheses 1 and 2. All regression coefficients were significant, except that of affective rumination on learning behavior (*β* = −0.10, *t* = −1.90, *ns*). Therefore, except Hypothesis 1c, Hypotheses 1a and 1b, and three sub-hypotheses of Hypothesis 2, were supported.

As shown in Model 2 and Model 4 of Table 3, cognitive job insecurity was significantly and positive associated with cognitive rumination (*β* = 0.37, *t* = 7.06, *p* < 0.01), while interpersonal conflict was significantly and positive associated with affective rumination (*β* = 0.51, *t* = 12.28, *p* < 0.01), lending support for Hypotheses 3 and 4. Although not formally hypothesized, results show that cognitive job insecurity was significantly and negatively related to affective rumination (*β* = −0.22, *t* = −5.16, *p* < 0.01, Model 4 in Table 3).

Next, we will examine the mediating effects of two forms of ruminations (i.e., Hypotheses 5 and 6). Since affective rumination was not significantly related to learning behavior (*β* = −0.10, *t* = −1.90, *p* = 0.059, Model 9 in Table 4), it did not mediate the effect of interpersonal conflict on learning behavior. Thus, Hypothesis 5c was not supported. Except for this effect, all other mediating effects were consistent with expectations of Hypotheses 5 and 6. Specifically, as shown in Table 4, after the independent variables (i.e., the two types of stressors) and the mediator variables (i.e., the two types of rumination) were included in the same regression equation (Models 3, 6, and 9 in Table 4), regression coefficients of the independent variables on the outcome variables were no longer significant or significantly decreased.

To vividly depict the interrelations among stressors, ruminations, and outcome variables, we provide a figure based on the results of hierarchical regression analyses.

We further used Hayes’ PROCESS macro [56] and constructed bias-corrected confidence intervals to formally test the significance of each indirect effect as stated in Hypotheses 5 and 6. Any indirect effect whose 95% confidence intervals exclude zero is statistically significant [56]. As shown in Table 5, affective rumination mediated effects of interpersonal conflict on negative affective (indirect effect = 0.149, 95% CI = [0.094, 0.209]) and positive affective (indirect effect = −0.081, 95% CI = [−0.144, −0.019]), but not on learning behavior (indirect effect = −0.041, 95% CI = [−0.085, 0.004]). Thus, Hypotheses 5a and 5b, but not Hypothesis 5c, were supported. On the other hand, cognitive rumination mediated effects of cognitive job insecurity on positive affective (indirect effect = 0.081, 95% CI = [0.045, 0.127]) and learning behavior (indirect effect = 0.111, 95% CI = [0.074, 0.154]), but not that on negative affective (indirect effect = −0.023, 95% CI = [−0.052, 0.003]), lending supports for Hypotheses 6b and 6c, but not 6a. Although not hypothesized, affective rumination could also mediate the effects of cognitive job insecurity on negative affective (indirect effect = −0.056, 95% CI = [−0.090, −0.029]) and positive affect (indirect effect = 0.030, 95% CI = [0.006, 0.062]).

## 4. Discussion

**Figure 2:**
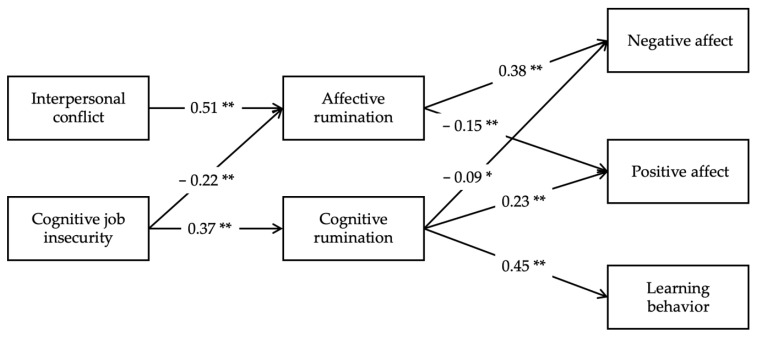
Differential mediating effects of affective and cognitive ruminations. Regression coefficients were taken from Table 3 and Table 4. Only those significant coefficients among key variables were retained. For brevity, all control variables were dropped from the figure. Note: * *p* < 0.05, ** *p* < 0.01.

Adopting the information processing perspective [13,14,22,23], our current study explores whether affective rumination and cognitive rumination are shaped by different antecedents, and whether these two forms of ruminations can result in differential consequences. Consistent with the dual-process theory of cognitive processing [29], results of the two-wave survey generally provide empirical evidence for our speculations. Specifically, as depicted in Figure 2, affective rumination is triggered by interpersonal conflict, a type of hindrance stressor that mostly hinders an individual’s well-being and development [38]. On the contrary, cognitive rumination is only elicited by cognitive job insecurity that belongs to a challenge stressor which is challenging but motivating and beneficial for personal growth [39,40]. Consistent with our hypotheses, the effects of affective and cognitive ruminations on individuals’ affective states are exact in opposite directions. Specifically, an individual’s affective rumination leads to stronger negative affect and reduces positive affect. In contrast, cognitive rumination can mitigate negative affect and enhance positive affect. Furthermore, cognitive rumination stimulates learning behavior, whereas affective rumination does not. Therefore, our study also provides evidence indicating that affective and cognitive ruminations can yield differential effects on individuals’ well-being and behavior.

It is particularly worthy of note that two forms of ruminations may function differently in channeling effects of stressors on individuals’ well-being and behavior. While affective rumination serves as the mechanism in transmitting the detrimental effects of a hindrance stressor (i.e., interpersonal conflict) on an individual’s affective states, cognitive rumination is effective in transferring the positive effects of a challenge stressor (i.e., cognitive job insecurity) to people’s affects and learning behavior. To summarize, the present study provides compelling evidence that affective and cognitive ruminations are of differential antecedents and consequences.

### 4.1. Theoretical Contributions

Our study has several important contributions for ruminations research. First of all, we introduce a novel perspective, that is, the information processing perspective, to the rumination literature. It has long been held that repetitive thinking only results in negative effects [5]. Recent studies have started to knowledge that rumination can be constructive or adaptive and can generate positive outcomes [13,14] if an individual engages in rumination in response to a positive affect [15,17] or to positive personal experiences [18], or learn to master the skills of active rumination [57]. Departing from these views, our current study highlights the important role of the information processing mode that people adopt in dealing with stressful events. Inspired by this novel perspective, we propose and provide empirical evidence for the argument that what consequences ruminative thinking may induce depend on the focus of information processing. For example, our study demonstrates that affective rumination only results in negative effects, probably because it is exclusively focused on one’s negative feelings. However, if people can switch to a cognitive mode by paying attention to the characteristics of events, what solutions they can have, or what their strengths and weaknesses are, they can benefit from this cognitive rumination. While our study provides some preliminary evidence for the differentiation of affective versus cognitive ruminations from the information processing perspective, more research is necessary to explore how the two forms of ruminations differ in terms of antecedents, consequences, underlying mechanisms, and boundary conditions.

Another contribution is that we integrate the dual-process theory of information processing [29] with the framework of challenge-hindrance stressors [38] to show that affective and cognitive ruminations can be triggered by different types of events. By doing so, we provide preliminary evidence for the nomological networks of affective and cognitive ruminations to account for the reason why two forms of ruminations are differentially associated with diverse antecedents and consequences. For example, our results indicate that hindrance stressors (i.e., interpersonal conflict) stimulate affective rumination, and consequently lead to lower affective well-being. On the contrary, challenge stressors (i.e., cognitive job insecurity) can stimulate cognitive rumination, and in turn bring in better affective well-being and more learning behavior. Unexpectedly, challenge stressors can also help individuals maintain better affective well-being by inhibiting affective rumination. Given that this is only a preliminary study, more research is necessary to reveal how affective and cognitive ruminations are differentially activated and how they yield differential effects on individuals’ well-being and behavior.

### 4.2. Practical Implications

People can use the current findings to take action to enhance the benefit from rumination and reduce the negative impact in a number of ways. First of all, more knowledge about rumination can enhance an individual’s self-awareness about what kind of rumination they are currently engaging in, then shift the focus from emotions to recognitive thinking. Second, it is possible to reduce the level of affective rumination by creating a harmonious working environment, such as establishing a transparent and fair work distribution and reward mechanism to reduce interpersonal conflicts. Finally, one can enhance the level of cognitive rumination by creating challenge stressors, such as setting up a challenge and an achievable goal and providing support for development.

### 4.3. Limitations and Future Directions

As always, our study has several limitations. First, our study used a two-wave strategy to collect survey data. Although this strategy is effective in mitigating common method variance [46], our study is still cross-sectional in nature and cannot address the causality between variables. There may exist an opposite causal relationship. For example, it is possible that negative emotional feelings can trigger subsequent affective ruminations, not the other way around. Similarly, it is possible that engaging in learning activities can stimulate more cognitive rumination. Future studies should adopt more rigorous designs, such as longitudinal ones, to explore how ruminations are triggered by and trigger other activities, events and behaviors. Second, given that ruminative thinking is highly variable, future studies should consider time-sensitive research designs, such as the experience sampling method, to better capture the dynamic characteristics of ruminations. Third, we only measured two types of stressors in the present study. It should be noted that people may encounter multiple stressful events in daily life and work. Moreover, even when facing with the same stressful event, different people can react in completely different ways. Thus, we encourage future studies to explore how different types of stressful events may trigger idiosyncratic ruminations and result in idiosyncratic consequences. Lastly, although we preliminarily constructed the nomological networks of affective and cognitive ruminations, we only examined a limited number of antecedents and consequences. Researchers can enrich the nomological networks by including more relevant constructs. For example, a recent study indicates that resilience is negatively correlated with affective rumination [58]. It will be interesting to explore whether resilience is positively related to cognitive rumination. Future studies should further explore the extent to which these two types of ruminations are related to and discriminated from other personal traits.

## 5. Conclusions

Guided by the dual-process theory of cognitive processing, the present study differentiates two forma of ruminations, affective rumination and cognitive rumination, respectively. A two-wave survey provides support for the argument that affective and cognitive ruminations are of differential antecedents and consequences. Affective rumination is triggered by the hindrance stressor (i.e., interpersonal conflict) and in turn transmits the negative effect of the latter on the individual’s affective well-being. On the contrary, cognitive rumination is elicited by the challenge stressor (i.e., cognitive job insecurity) and translates its positive effect on affective well-being and learning behavior.

## Figures and Tables

**Table 1:** Results of confirmatory factor analyses.

Models	χ^2^ (df) ^a^	χ^2^/df	Δχ^2^ (Δdf) ^a^	CFI	TLI	RMSEA	SRMR
Seven-factor model (Hypothesized model)	870.51 (384)	2.27	/	0.92	0.90	0.056	0.06
2. Six-factor model 1: Interpersonal conflict and cognitive job insecurity combined as one factor	1201.15 (390)	3.08	330.64 (6)	0.86	0.84	0.071	0.07
3. Six-factor model 2: Cognitive and affective rumination combined as one factor	1465.82 (390)	3.76	595.31 (6)	0.81	0.79	0.082	0.11
4. Six-factor model 3: Interpersonal conflict and affective rumination combined as one factor	1015.89 (390)	2.60	145.38 (6)	0.89	0.88	0.063	0.05
5. Six-factor model 4: Cognitive job insecurity and cognitive rumination combined as one factor	1256.32 (390)	3.22	385.81 (6)	0.85	0.83	0.074	0.08
6. Six-factor model 5: Affective rumination and negative affect combined as one factor	1298.36 (390)	3.33	427.85 (6)	0.84	0.82	0.075	0.08
7. Six-factor model 6: Affective rumination and positive affect combined as one factor	1532.74 (390)	3.93	662.23 (6)	0.80	0.78	0.085	0.09
8. Six-factor model 7: Cognitive rumination and learning behavior combined as one factor	1023.41 (390)	2.62	152.90 (6)	0.89	0.88	0.063	0.07
9. Six-factor model 1: Positive and negative affects combined as one factor	1204.01 (390)	3.09	333.50 (6)	0.86	0.84	0.071	0.07
10. Single-factor model: All variables collapsed as a single construct	2956.57 (405)	7.30	2086.06 (11)	0.55	0.52	0.124	0.11

Note. ^a^: Each χ^2^ or Δχ^2^ was significant at the level of *p* < 0.001. CFI = Comparative Fit Index; TLI = Tucker–Lewis index; RMSEA = Root Mean Square Error of Approximation; SRMR = Standardized Root Mean Square Residual. Δχ^2^ of each six-factor model or the single-factor model was calculated and tested against the hypothesized seven-factor model.

**Table 2:** Means, standard deviations, and correlation coefficients of each variable.

Variable	Mean	SD	1	2	3	4	5	6	7
Interpersonal conflict	2.49	0.6	(0.79)						
2. Cognitive job insecurity	3.89	0.76	−0.42 **	(0.82)					
3. Affective rumination	2.35	0.75	0.61 **	−0.45 **	(0.87)				
4. Cognitive rumination	3.95	0.56	−0.08	0.36 **	−0.10 *	(0.76)			
5. Learning behavior	4.09	0.45	−0.27 **	0.44 **	−0.29 **	0.54 **	(0.80)		
6. Positive affect	3.75	0.6	−0.34 **	0.49 **	−0.38 **	0.38 **	0.56 **	(0.87)	
7. Negative affect	1.72	0.45	0.38 **	−0.44 **	0.52 **	−0.22 **	−0.37 **	−0.54 **	(0.86)

Note: *n* = 410. The number in parentheses on the diagonal was the internal consistency coefficient α for each variable. * *p* < 0.05, ** *p* < 0.01.

**Table 3:** Regression analysis results of the effects of stressors on affective and cognitive ruminations.

Variable	DV: Cognitive Rumination		DV: Affective Rumination	
	Model 1	Model 2	Model 3	Model 4
Gender	0.01	−0.01	−0.09	−0.03
Age	0.30 **	0.18 *	−0.18 *	−0.09
Job tenure	−0.30 **	−0.19 **	0.02	−0.03
Interpersonal conflict		0.07		0.51 **
Cognitive job insecurity		0.37 **		−0.22 **
*R* ^2^	0.05	0.16	0.04	0.44
*F*	*F* (3,41) = 6.40 ***	*F* (5,40) = 14.89 ***	*F* (3,40) = 6.03 **	*F* (5,40) = 62.16 ***
Δ*R*^2^		0.11		0.40
Δ*F*		*F* (2,40) = 26.42 ***		*F* (2,40) = 140.15 ***

Note: *n* = 490. Gender: 0 = male, 1 = female. Standardized regression coefficients were shown in the table. * *p* < 0.05, ** *p* < 0.01, *** *p* < 0.001.

**Table 4:** Regression analysis results of the mediating effects of affective and cognitive ruminations.

Variable	DV: Positive Affect			DV: Negative Affect			DV: Learning Behavior		
	Model 1	Model 2	Model 3	Model 4	Model 5	Model 6	Model 7	Model 8	Model 9
Gender	0.05	0.01	0.01	−0.01	0.03	0.04	0.02	−0.02	−0.02
Age	0.40 **	0.27 **	0.21 **	−0.25 **	−0.13	−0.08	0.29 **	0.16 *	0.08
Job tenure	−0.33 **	−0.24 **	−0.20 **	0.15 *	0.07	0.07	−0.21 **	−0.12	−0.03
Interpersonal conflict		−0.18 **	−0.13 *		0.25 **	0.06		−0.12 *	−0.10 *
Cognitive job insecurity		0.37 **	0.26 **		−0.32 **	−0.20 **		0.36 **	0.18 **
Affective rumination			−0.15 **			0.38 **			−0.10
cognitive rumination			0.23 **			−0.09 *			0.45**
*R* ^2^	0.08	0.3	0.35	0.03	0.25	0.34	0.04	0.21	0.39
*F*	*F*(3,41) = 11.11 ***	*F*(5,40) = 33.80 ***	*F*(7,40) = 30.99 ***	*F*(3,41) = 3.967 **	*F*(5,40) = 26.76 ***	*F*(7,40) = 29.17 ***	*F*(3,41) = 5.44 **	*F*(5,40) = 21.86 ***	*F*(7,40) = 36.56 ***
Δ*R*^2^		0.22	0.06		0.22	0.09		0.17	0.18
Δ*F*		*F*(2,40) = 62.78 ***	*F*(2,40) = 17.18 ***		*F*(2,40) = 59.24 ***	*F*(2,40) = 26.70 ***		*F*(2,40) = 44.74 ***	*F*(2,40) = 57.92 ***

Note: *n* = 410. Gender: 0 = male, 1 = female. Standardized regression coefficients were shown in the table. * *p* < 0.05, ** *p* < 0.01, *** *p* < 0.001.

**Table 5:** Indirect effects of affective and cognitive ruminations.

Indirect Path	Indirect Effect	Boot SE	95% Boot CI
Interpersonal conflict → affective rumination → negative affect (H5a)	0.149	0.029	[0.094, 0.209] *
2. Interpersonal conflict → affective rumination → positive affect (H5b)	−0.081	0.032	[−0.144, −0.019] *
3. Interpersonal conflict → affective rumination → learning (H5c)	−0.041	0.023	[−0.085, 0.004]
4. Cognitive job insecurity → cognitive rumination → negative affect (H6a)	−0.023	0.014	[−0.052, 0.003]
5. Cognitive job insecurity → cognitive rumination → positive affect (H6b)	0.081	0.021	[0.045, 0.127] *
6. Cognitive job insecurity → cognitive rumination → learning (H6c)	0.111	0.020	[0.074, 0.154] *
7. Cognitive job insecurity → affective rumination → negative affect	−0.056	0.016	[−0.090, −0.029] *
8. Cognitive job insecurity → affective rumination → positive affect	0.030	0.015	[0.006, 0.062] *
9. Cognitive job insecurity → affective rumination → learning	0.015	0.001	[−0.001, 0.037]

Note. * The 95% boot CI did not include zero, indicating a significant indirect effect.

## Data Availability

Data are available upon request from the corresponding author. The data are not publicly available due to privacy or ethical restrictions.
